# Risk of Tumor Upstaging With Prostate-Specific Membrane Antigen Positron Emission Tomography in Patients With High-Risk Prostate Cancer

**DOI:** 10.1001/jamanetworkopen.2022.31101

**Published:** 2022-09-12

**Authors:** Aaron Brant, Patrick Lewicki, Michael Xiang, Alec Zhu, Amar U. Kishan, Erqi Liu Pollom, Jonathan E. Shoag

**Affiliations:** 1 Department of Urology, New York-Presbyterian Hospital, Weill Cornell Medicine, New York; 2 Department of Radiation Oncology, UCLA Medical Center, Los Angeles, California; 3 Department of Radiation Oncology, Stanford University School of Medicine, Palo Alto, California; 4 Department of Radiation Oncology, Palo Alto Veterans Health Care System, Palo Alto, California; 5 Department of Urology, University Hospitals Cleveland Medical Center, Case Western Reserve University School of Medicine, Cleveland, Ohio

## Abstract

This cross-sectional study of data from the National Comprehensive Cancer Network assesses the association of prostate-specific membrane antigen positron emission tomography with tumor upstaging risk in patients with high-risk prostate cancer.

## Introduction

Prostate-specific membrane antigen positron emission tomography (PSMA PET) was recently approved by the Food and Drug Administration^1^ and incorporated into the National Comprehensive Cancer Network (NCCN) guidelines.^2^ With evidence that PSMA PET has improved sensitivity in detecting nodal and metastatic disease compared with conventional imaging,^3^ wider use of PSMA PET will result in more patients found to have otherwise occult nodal or metastatic disease.

Current evidence and guidelines recommend radiation therapy with androgen deprivation therapy as the preferred treatment for prostate cancer with node-positive disease detected by conventional imaging.^4,5^ However, it is unknown whether men with positive nodes detectable by PSMA PET but undetectable by conventional imaging benefit preferentially from radiotherapy. We used a validated risk calculator to quantify the association of routine PSMA PET use with risk of tumor upstaging in patients who had undergone surgery.^6^

## Methods

This study was approved by the University of California, Los Angeles Institutional Review Board and was deemed exempt from review because of the use of deidentified data. In this cross-sectional analysis, the UCLA PSMA Risk Calculator, a validated nomogram for calculating the likelihood of upstaging on [^68^Ga]Ga-PSMA PET, was applied to patients in the National Cancer Database (NCDB) who underwent radical prostatectomy from 2010 to 2017 and were identified as having clinically localized high-risk disease according to NCCN guidelines.^4^ The current analysis was performed between December 1, 2021, and January 1, 2022. Nomogram inputs, consisting of clinical T stage, prostate-specific antigen (PSA) level, Gleason grade group, and percentage of positive cores on systematic biopsy,^6^ were extracted from the NCDB as independent variables. The median (IQR) risk (as a percentage) of PSMA PET upstaging probability was then calculated along with changes in nomogram inputs over time. The Jonckheere-Terpstra test was applied to determine the significance of the trend. Statistical analysis was performed in MATLAB, version R2020a (MathWorks, Inc). This study followed the Strengthening the Reporting of Observational Studies in Epidemiology (STROBE) reporting guideline.

## Results

Out of 352 521 patients who underwent surgery, 207 713 patients were identified after excluding patients with missing T stage, Gleason grade group, PSA level, or biopsy core data. A total of 45 772 men with high-risk disease were identified. The median (IQR) age was 64 years (59-68) years, the median (IQR) PSA level was 8.8 ng/mL (5.6-21.3 [to convert to micrograms per liter, multiply by 1.0]), the median (IQR) percentage of positive cores was 50% (26%-75%), and the most frequent Gleason grade groups were 4 and 5 in 21 293 (46.5%) and 13 750 (30.0%) individuals, respectively.

**Figure 1.**
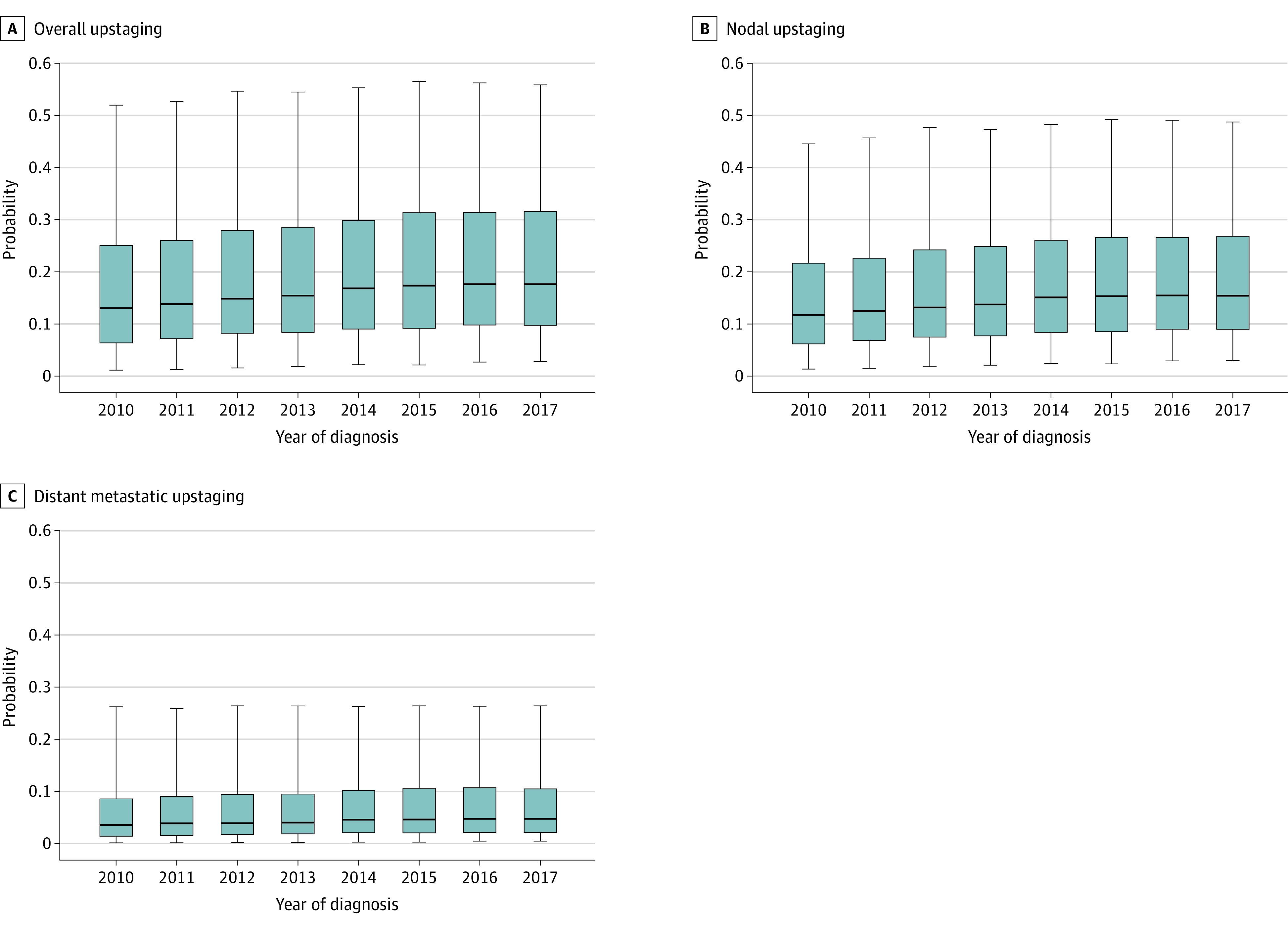
Probability Distribution of Overall Nodal and Distant Metastatic Upstaging on Prostate-Specific Membrane Antigen Positron Emission Tomography Probability was calculated using the University of California, Los Angeles, Prostate-Specific Membrane Antigen Risk Calculator among clinically localized high-risk patients undergoing radical prostatectomy in the National Cancer Database, over time. The horizontal bars represent the median, boxes represent IQRs, and whiskers demarcate the upper and lower 5% of patients.

**Figure 2.**
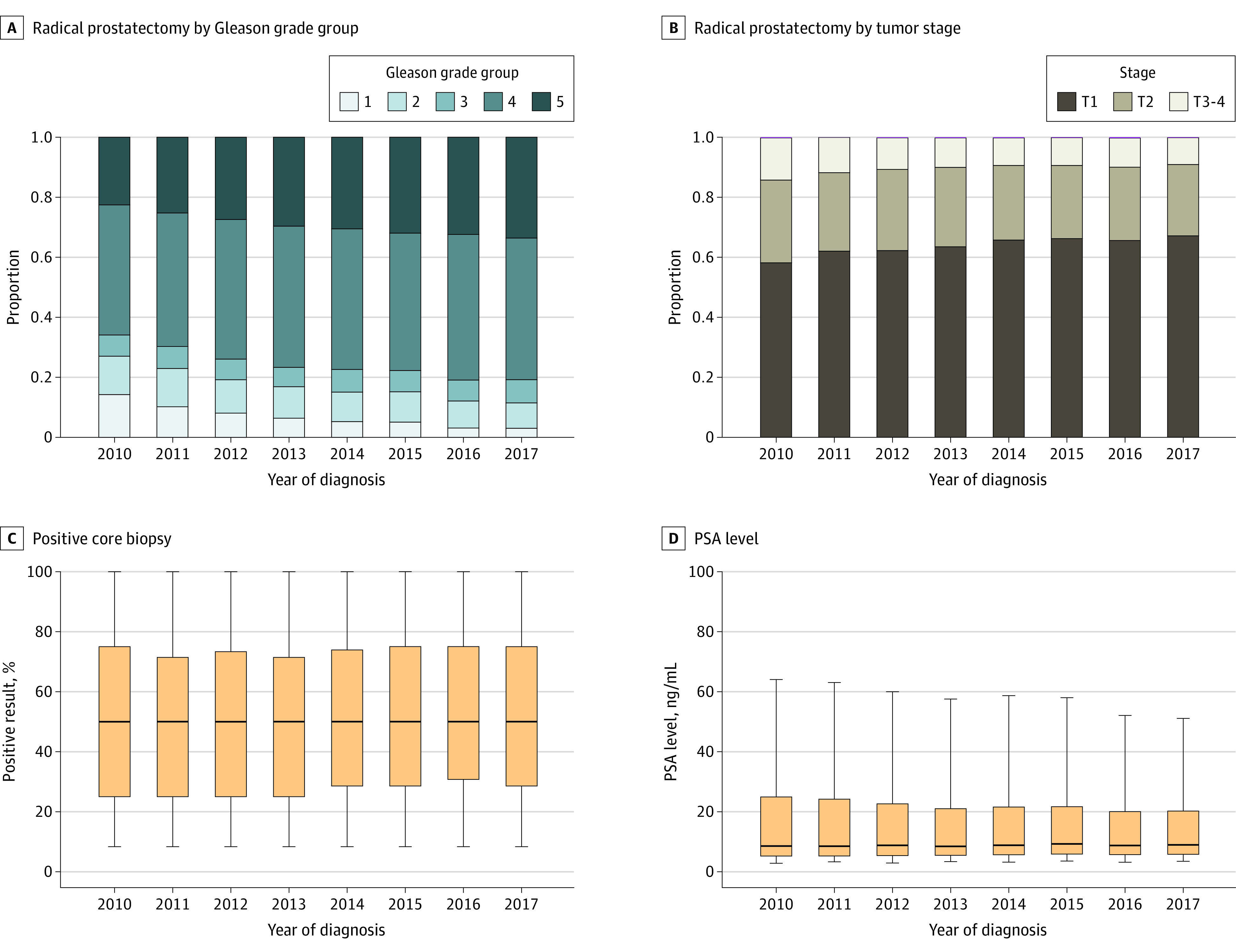
Proportions of Gleason Grade Group and Clinical T Stage Over Time Among Patients Who Underwent Radical Prostatectomy for Clinically Localized High-Risk Prostate Cancer C and D, The horizontal bars represent the median, boxes represent IQRs, and whiskers demarcate the upper and lower 5% of patients. To convert prostate specific antigen (PSA) from ng/mL to μg/L, multiply by 1.0.

The median (IQR) risk of PSMA PET upstaging was 16.3% (8.5%-29.6%) from 2010 to 2017, which increased year by year, from 13.0% in 2010 to 17.6% in 2017 (*P* < .001) (Figure 1). The risk of nodal and distant metastatic upstaging significantly increased over time, from 11.7% in 2010 to 15.4% in 2017 (*P* < .001) and from 3.6% in 2010 to 4.7% in 2017 (*P* < .001), respectively. While PSA, T stage, and the percentage of positive cores did not increase over time, the proportion of Gleason grade group 4 and 5 cancers did (Figure 2).

## Discussion

With application of a validated risk calculator to a national cohort of patients with high-risk prostate cancer, the estimated median risk of upstaging from PSMA PET use within the entire cohort was 16.3%, suggesting that the risk of upstaging has steadily increased over time, a trend that may be secondary to higher proportions of Gleason grade group 4 and 5 cancers being found after surgery. Limitations exist in the true predictive accuracy of the risk calculator, given its receiver operative curve of 0.74.^6^ While upstaging may be secondary to nodal or distant metastatic findings on PSMA PET, the risk of nodal upstaging in particular may affect the decision between surgery and radiation. Therefore, with increasing risk of nodal upstaging, the immediate impact of stage migration from PSMA PET may be to alter treatment decisions in a substantial number of patients with high-risk prostate cancer.
